# Apoptosis and Necrosis: Two Types of Cell Death in Alcoholic Liver Disease

**Published:** 1997

**Authors:** Amin A. Nanji, Susanne Hiller-Sturmhöfel

**Affiliations:** Amin A. Nanji, M.D., is co-director of the Center for Clinical and Laboratory Investigation, Department of Pathology, Beth Israel Deaconess Medical Center, and associate professor of pathology at Harvard Medical School, Boston, Massachusetts. Susanne Hiller-Sturmhöfel, Ph.D., is a science editor of Alcohol Health & Research World

**Keywords:** alcoholic liver disorder, necrosis, cytolysis, hepatocyte, cytochrome P450, oxidation-reduction, iron, metabolic disorder, ATP (adenosine triphosphate), hypoxia, endotoxins, biochemical mechanism, pathogenesis, literature review

## Abstract

Heavy alcohol consumption over long periods of time can result in severe liver damage, including death of liver cells (i.e., hepatocytes). Two mechanisms—apoptosis and necrosis—can contribute to hepatocyte death. In apoptosis, the affected cell actively participates in the cell death process, whereas in necrosis the cell death occurs in response to adverse conditions in the cell’s environment. Numerous factors that may contribute to the initiation of hepatocyte apoptosis are affected by alcohol consumption. These factors include the enzyme cytochrome P450 2E1 (i.e., CYP2E1), small molecules (i.e., cytokines) involved in cell communication, oxidative stress, and changes in iron metabolism. Similarly, alcohol consumption can influence several factors believed to be involved in hepatocyte necrosis, including depletion of the energy-storing molecule adenosine-triphosphate, reduced oxygen levels (i.e., hypoxia) in the liver, oxidative stress, and bacterial molecules called endotoxins.

Many people who drink heavily over extended periods of time (i.e., several years) develop increasingly severe liver damage, including fatty liver, alcoholic hepatitis, and alcoholic cirrhosis. Fatty liver is caused by the accumulation of fat in the liver. Alcoholic hepatitis is characterized by extensive inflammation of the liver and the destruction of liver cells (i.e., hepatocytes). Moreover, scar tissue begins to form, replacing healthy liver tissue. In alcoholic cirrhosis, scarring and cell death progress further, resulting in distortion of the internal structure of the liver and, subsequently, in severe functional impairment and secondary failure of other organs, such as the kidney. These multiple complications can lead to the patient’s death. By investigating the mechanisms underlying alcohol’s deleterious effects on the liver, researchers hope to ultimately develop new diagnostic and therapeutic approaches to prevent these often fatal consequences of alcohol consumption.

**Figure:**
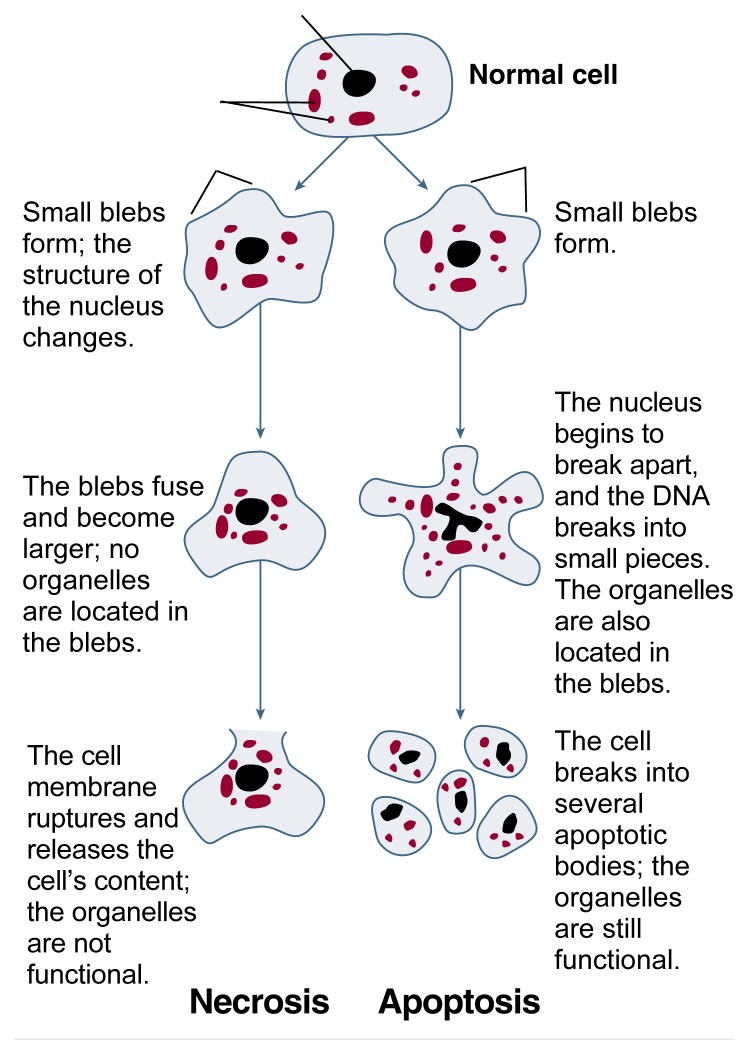
Structural changes of cells undergoing necrosis or apoptosis.

Much recent research has focused on the mechanisms that contribute to hepatocyte death at the cellular level. Two processes play a role in hepatocyte destruction—apoptosis and necrosis. This article briefly reviews the differences between these two processes and speculates on some of their underlying mechanisms. The article also discusses how heavy alcohol consumption may be associated with the mechanisms that promote these processes.

## Differences Between Apoptosis and Necrosis

Although the ultimate results of apoptosis and necrosis are the same (i.e., death of the affected cells), the two processes differ significantly. In apoptosis, the affected cells actively participate by activating a cascade of biochemical reactions that result in cell death. Accordingly, apoptosis has been called cell suicide (e.g., Rosser and Gores 1995).^1^ In necrosis, however, cell death occurs because of adverse conditions or changes in the cell’s environment. Thus, necrosis can be viewed as the consequence of a “biological accident” that leads to the death of an “innocent victim” (Rosser and Gores 1995).

**Table:** Factors That May Contribute to Hepatocyte Apoptosis and Necrosis in Alcoholic Liver Disease

Factor	Research Findings Regarding Alcohol’s Effects on These Factors
***Apoptosis***	
Cytochrome P450 2E1 (CYP2E1)	Alcohol metabolism by CYP2E1 results in the generation of oxygen radicals.
Cytokines	Patients with alcoholic hepatitis and rats with alcohol-induced liver injury show elevated levels of tumor necrosis factor alpha (TNF-α). Chronic alcohol consumption increases the levels of TNF-α receptors on the hepatocytes. Chronic alcohol consumption induces the production of transforming growth factor-beta 1(TGF-β1).
Iron metabolism	Chronic alcohol consumption can increase iron levels in the body and levels of free iron in the liver.
Oxidative stress	Alcohol metabolism by CYP2E1 and increased levels of free iron increase the levels of oxygen radicals.

***Necrosis***	
ATP depletion	Patients with alcoholic hepatitis have reduced levels of ATP in their cells.
Cytokines	Patients with alcoholic hepatitis and rats with alcohol-induced liver injury show elevated levels of TNF-α. Chronic alcohol consumption increases the levels of TNF-α receptors on the hepatocytes. Chronic alcohol consumption induces the production of TGF-β1. Other inflammatory cytokines, such as interleukin-8, are increased in alcoholic hepatitis.
Hypoxia	Alcohol metabolism results in increased oxygen consumption by the liver.
Oxidative stress	Alcohol metabolism by CYP2E1 and increased levels of free iron increase the levels of oxygen radicals. Rats receiving an alcohol-containing diet have reduced glutathione levels in their mitochondria. Patients with alcoholic liver disease have reduced levels of vitamin E.
Endotoxin	In alcoholics, the number of endotoxin-producing bacteria in the intestine is elevated. In addition, endotoxin can enter the bloodstream more easily, and Kupffer cells have a reduced capacity to detoxify endotoxin.

NOTE: For definitions of terms, see glossary, pp. 330.

Characteristic differences also exist in both the structure and the metabolic processes of cells that undergo apoptosis or necrosis (see figure, p. 325) (Rosser and Gores 1995). When a cell undergoes apoptosis, the entire cell, including the nucleus, separates into numerous fragments (i.e., apoptotic bodies). Simultaneously, the genetic material (i.e., DNA) of apoptotic cells breaks into a characteristic pattern of pieces of varying sizes. During the breakup of the cell, the cell continues to produce proteins and adenosine triphosphate (ATP), a molecule that is required for most of the cell’s energy-consuming metabolic processes and which is essential for cell functioning. As a result, each apoptotic body, which is surrounded by a piece of cell membrane, contains intact, functional cell components (i.e., organelles^2^).

Necrotic cell death, in contrast, is characterized by the loss of metabolic functions and of the integrity of the cell membrane. Thus, cells undergoing necrosis cease their production of proteins and ATP. Structurally, the cells’ organelles swell and become nonfunctional during the initial stages of necrosis. In addition, the cell membrane forms bubblelike projections (i.e., blebs). These blebs, which contain no organelles, fuse and increase in size. Eventually, the cell membrane ruptures, resulting in the release of the cell’s components into the surrounding tissue. This process of cell dissolution is called cytolysis. The released cellular content subsequently induces an inflammatory response in the effected tissue (e.g., the liver). This response is mediated by three components: (1) certain cells of the immune system that are attracted to the liver; (2) small molecules called cytokines that are involved in cell communication; and (3) reactive oxygen species (i.e., oxygen radicals). This subsequent inflammatory response, which often is considered an integral part of necrosis, further damages the liver tissue.

## Causes of Apoptosis and Necrosis

Numerous factors and mechanisms can induce apoptotic and necrotic hepatocyte death. Some of these factors and mechanisms contribute to both apoptosis and necrosis, whereas others play a role in only one of these processes (see table, p. 326). Chronic alcohol consumption may induce or exacerbate many of these mechanisms.

### Contributing Factors to Apoptosis

Among the numerous factors that can contribute to hepatocyte apoptosis, the following may be particularly relevant in alcoholic liver disease:

- The enzyme cytochrome P450 2E1 (i.e., CYP2E1)
- Cytokines, such as tumor necrosis factor-alpha (TNF-α) and transforming growth factor-beta 1 (TGF-β1)
- Oxidative stress
- Altered iron metabolism.

#### CYP2E1

The liver is the primary site of alcohol metabolism. Several pathways for alcohol metabolism exist in hepatocytes. One of these pathways involves a group of enzymes collectively known as cytochrome P450, which primarily serves to detoxify harmful substances. One of these enzymes is CYP2E1. Alcohol metabolism by CYP2E1 results in the formation of highly reactive, oxygen-containing molecules called oxygen radicals. Oxygen radicals are chemically or electrically unstable; therefore, they quickly interact with other molecules in the cells, such as fat molecules (i.e., lipids), proteins, and DNA. For example, oxygen radicals can interact with the lipids that comprise the membranes within and surrounding the cells. This process is called lipid peroxidation. As a result of lipid peroxidation, the membranes gradually are degraded. This loss of membrane integrity severely impairs cell function, and the cells eventually die through an apoptotic mechanism.

Researchers have demonstrated that the CYP2E1-dependent pathway of alcohol-induced lipid peroxidation and subsequent hepatocyte apoptosis occurs in rats receiving a high-fat diet (i.e., a diet rich in lipid components called polyunsaturated fatty acids) plus alcohol (Nanji et al. 1994*a*,*b*). Other investigators have evaluated the relationship among CYP2E1, unsaturated fatty acids, lipid peroxidation, and apoptotic cell death by exposing a hepatocyte cell line that overproduces CYP2E1 to the unsaturated fatty acid arachidonic acid (Chen et al. 1997). This treatment resulted in lipid peroxidation and hepatocyte apoptosis. Substances that prevented the formation of oxygen radicals by CYP2E1 or that eliminated, or scavenged, these radicals (i.e., antioxidants) prevented apoptotic hepatocyte death. These observations further support the hypothesis that alcohol-induced activity of CYP2E1 may contribute to alcohol-induced liver injury.

#### Cytokines

Cytokines are small molecules secreted primarily by cells of the immune system in order to communicate with other immune cells and cells outside the immune system. (For more information on cytokines and their role in alcoholic liver disease, see the article by McClain et al., pp. 317–320.) Two cytokines that can induce apoptosis are TNF-α and TGF-β1.^3^

In the liver, TNF-α is produced and secreted by a certain type of immune cell called Kupffer cells. Several studies have found that TNF-α levels were elevated in patients with alcoholic hepatitis and in rats with alcohol-induced liver injury (McClain et al. 1993; Nanji et al. 1994*c*). To exert its effects on the cell, TNF-α must interact with specific docking molecules (i.e., receptors) on the cell’s surface. Several types of TNF-α receptors exist. Hepatocytes carry receptors that respond even to low TNF-α concentrations. Thus, hepatocytes are naturally sensitive to the cytokine. Chronic alcohol consumption increases the number of these receptors on the hepatocytes, thereby enhancing the cells’ sensitivity to TNF-α even further (Deaciuc et al. 1995). In addition, hepatocytes carry a receptor called CD95, or Fas, which is similar to the TNF-α receptors. This receptor interacts with a specific molecule (i.e., Fas ligand) found on the surface of certain immune cells (i.e., cytotoxic T cells). The interaction of the Fas ligand and Fas receptor initiates chemical processes in the cell that lead to apoptosis. In alcoholic patients, high numbers of cytotoxic T cells accumulate in the liver (Chedid et al. 1993), suggesting that these cells, through the Fas ligand-Fas interaction, also may contribute to hepatocyte apoptosis in these patients.

The second cytokine implicated in hepatocyte apoptosis, TGF-β1, is produced by Kupffer cells and by stellate cells, a cell type involved in the formation of scar tissue during alcoholic liver disease. (For more information on stellate cells, see the article by Friedman, pp. 310–316.) In rats, chronic alcohol consumption induces TGF-β1 production by Kupffer and stellate cells (Maher 1997). Furthermore, studies in cultured hepatocytes found that TGF-β1 induces apoptosis in these cells (Oberhammer et al. 1992). Thus, increased TGF-β1 production after alcohol consumption may promote hepatocyte apoptosis.

#### Oxidative Stress

Oxidative stress results from the excessive generation of oxygen radicals and/or the lack of antioxidants (e.g., a substance called glutathione and vitamins A and E) to scavenge these radicals. Oxidative stress occurs in conjunction with the depletion of ATP and reduced glutathione levels in the cell. As described in the following section, oxidative stress is characteristically associated with necrosis. Under certain conditions, however, oxidative stress also may induce apoptosis (Slater et al. 1995). As discussed later in this article, alcohol also can induce oxidative stress and thereby may contribute to hepatocyte death by apoptosis as well as necrosis. (For more information on oxidative stress and its role in alcoholic liver disease, see the article by Fernández-Checa et al., pp. 321–324.)

#### Changes in Iron Metabolism

Excessive iron levels in the liver also may contribute to liver damage, including hepatocyte apoptosis. Some studies have suggested that free iron (i.e., iron not bound to proteins) promotes the formation of oxygen radicals, thereby increasing oxidative stress in the hepatocytes (Shaw 1989; Sadrzadeh et al. 1994*b*). Moreover, experiments in rats found that the accumulation of iron in the liver caused various changes in that organ, including hepatocyte apoptosis (Kato et al. 1996). Chronic alcohol consumption can increase iron levels in the body—for example, by enhancing iron absorption from the food in the gastrointestinal tract or by the excessive consumption of iron-rich alcoholic beverages, such as red wine (Ballard 1997). In fact, approximately 30 percent of alcoholics have excessive iron levels in their livers, and a substantial percentage of that iron is not bound to protein (Nanji and Zakim 1996). Thus, it is possible that these elevated levels of free iron may contribute to alcohol-induced hepatocyte apoptosis in alcoholic patients.

### Contributing Factors to Necrosis

As with apoptosis, numerous factors likely contribute to alcohol-induced hepatocyte necrosis. Some of these factors, such as the depletion of ATP, reduced oxygen levels (i.e., hypoxia) in the liver, and oxidative stress, directly cause cell damage. Other factors, such as endotoxins—toxic pieces of the cell walls of bacteria that live in the intestine—and some aspects of increased oxidative stress, play a more indirect role by initiating or exacerbating the inflammatory response associated with hepatocyte necrosis (Nanji and Zakim 1996).

#### ATP Depletion

As previously mentioned, ATP is vital to cell functioning because it provides the energy required for many cellular reactions. For example, ATP provides the energy for mechanisms to ensure that the correct concentrations of various charged particles (i.e., ions, such as sodium, potassium, or calcium) are maintained in the cell. Accordingly, ATP depletion rapidly leads to the breakdown of the cell’s ion balance. As a result, numerous cellular processes cannot function properly, and the cell dies. In fact, disturbances in the ion concentrations are often present during early stages of cell necrosis (Rosser and Gores 1995).

In patients with alcoholic hepatitis, the cells’ ATP levels are reduced. The extent of this reduction is correlated with the severity of the liver disease (Menon et al. 1995). Several mechanisms may contribute to alcohol-induced ATP depletion (Nanji and Zakim 1996; Nanji 1997). For example, ATP production, which occurs primarily in organelles called mitochondria, may be impaired. Alternatively, the cell’s ATP consumption may increase as the result of sodium imbalances in the cell.

#### Hypoxia

All cells, including hepatocytes, require oxygen for their metabolism, most importantly for ATP generation. Accordingly, hypoxia can lead to ATP depletion and, subsequently, to necrosis. Some researchers have suggested that alcohol metabolism results in increased oxygen use in the liver, thereby lowering the amount of oxygen available for other cellular functions (Maher 1997). Furthermore, studies in animal models of alcoholic liver disease found that the animals could not compensate for this increased oxygen consumption by enhancing oxygen delivery to the liver via the blood (Tsukamoto and Xi 1989). Hypoxia is particularly likely to occur in regions of the liver that normally are exposed to lower oxygen concentrations. Alcohol-induced liver damage also tends to occur primarily in those regions, supporting the association of hypoxia with alcoholic liver disease (Maher 1997).

#### Oxidative Stress

The presence of excess oxygen radicals and/or the lack of sufficient antioxidants to scavenge these radicals can either directly contribute to hepatocyte necrosis or enhance the inflammatory response associated with necrosis. Oxygen radicals appear to cause hepatocyte necrosis by modifying DNA, lipids, and proteins in the cells. As mentioned previously, oxygen radicals can induce lipid peroxidation, resulting in the destabilization of membranes within and around the cell. For example, lipid peroxidation of the membrane surrounding the mitochondria interferes with the generation of ATP in these organelles, thus contributing to ATP depletion. Similarly, the interaction of oxygen radicals with proteins could interfere with the proteins’ functions.

Chronic alcohol consumption can increase oxidative stress through several mechanisms (Nanji and Zakim 1996). For example, alcohol metabolism by the cytochrome P450 complex, specifically by CYP2E1, is associated with the generation of oxygen radicals. As discussed earlier, chronic alcohol consumption also increases the liver’s free iron levels, which can promote the generation of oxygen radicals. Moreover, alcohol promotes the generation of fatty acids, which can accumulate in the liver and serve as targets for lipid peroxidation.

In addition to increasing the levels of oxygen radicals, alcohol also can enhance oxidative stress by reducing the levels or activities of antioxidants, which could directly contribute to hepatocyte necrosis. These antioxidants include various enzymes (e.g., catalase, superoxide dismutase, and glutathione peroxidase) that eliminate oxygen radicals from the cell by converting them into nontoxic substances (e.g., water molecules). In animal models of alcoholic liver disease, the levels of all three of these enzymes were reduced, thereby increasing oxidative stress (Nanji 1997).

Nonprotein antioxidants include glutathione and vitamin E. Rats that were fed an alcohol-containing diet had significantly reduced glutathione levels in their mitochondria (García-Ruiz et al. 1994). Decreased glutathione levels result in the loss of mitochondrial function (e.g., ATP generation) and thereby may contribute to hepatocyte necrosis. Similarly, patients with alcoholic liver disease exhibit reduced vitamin E levels (Sadrzadeh et al. 1994*a*).

Oxidative stress also can contribute to the inflammatory reaction associated with necrosis. For example, in Kupffer cells, oxygen radicals can activate several genes that code for cytokines, such as TNF-α, which are involved in inflammatory reactions (Nanji 1997). Similarly, lipid peroxidation can increase the production of TNF-α and other inflammatory molecules.

#### Endotoxins

The outer cell walls of many bacteria (e.g., those living in the intestine) contain complex molecules made up of fat and sugar components (i.e., lipopolysaccharides). These molecules also are called endotoxins, because when the bacteria die, the lipopolysaccharide molecules are released and can enter the bloodstream, where they can cause fever, chills, shock, and other symptoms of an infection.

Studies in both humans and animals with alcoholic liver disease have found that both acute and chronic alcohol consumption can result in increased endotoxin levels in the blood (Nanji 1997). Moreover, in animal models of alcoholic liver disease, the severity of the liver damage was correlated with the endotoxin levels in the animals’ blood. Several mechanisms contribute to increased endotoxin levels in alcoholics. For example, the number of endotoxin-producing bacteria in the intestine is elevated. In addition, changes in the wall of the intestine allow endotoxins to enter the bloodstream more easily, and Kupffer cells in the liver have a reduced capacity to detoxify endotoxin molecules (Nanji 1997). Although Kupffer cells in the livers of alcoholics may eliminate endotoxin less efficiently, these cells still interact with the endotoxin molecules. This interaction is mediated by two proteins, lipopolysaccharide binding protein (LBP) and CD14. In animal models of alcoholic liver disease, the levels of LBP and CD14 in the liver were elevated and correlated with the presence of necrosis and/or inflammation of the liver (Nanji 1997). As a result of the interaction with endotoxin, the affected Kupffer cells increase their production of various molecules that initiate inflammatory and immune responses in the liver. These molecules may perpetuate the inflammation characteristic of alcoholic hepatitis.

## Conclusions

Alcohol consumption may contribute to the death of hepatocytes by apoptosis and necrosis through numerous mechanisms. Of these two processes, apoptosis occurs at all stages of alcoholic liver disease, whereas necrosis generally is found in advanced stages (i.e., alcoholic hepatitis and cirrhosis). As researchers learn more about the various mechanisms underlying apoptosis and necrosis and about alcohol’s role in these processes, this knowledge may eventually lead to new approaches to diagnosing, preventing, and treating alcoholic liver disease. For example, alcohol-induced hepatocyte necrosis may conceivably be prevented by increasing the levels of antioxidants or by decreasing the concentrations of oxygen radicals in the mitochondria. Researchers have prevented glutathione depletion in alcohol-fed animals by administering a glutathione precursor to the animals (Maher 1997). Similarly, dietary supplementation with antioxidants might become a useful tool in the treatment of alcoholic hepatitis (Rosser and Gores 1995). To date, however, such attempts have not yielded promising results.

## Footnotes

1 It is important to recognize that apoptosis is not always a deleterious process. Rather, it generally serves to eliminate damaged or malfunctioning cells from the body. Thus, apoptosis plays a vital role in ensuring the proper functioning of many organs (e.g., the immune system).

2 For a definition of this and other technical terms used in this article, see glossary, p. 330.

3 Depending on the environmental conditions of the cells receiving the TNF-α signal, the cytokine can have different effects (e.g., promote apoptosis or necrosis or stimulate cell growth).

## Abbreviations

Adenosine triphosphate (ATP): A molecule that stores energy and provides it for most energy-consuming processes in the cell.
Antioxidant: A substance that inhibits *oxidation*.
Collagen: The major protein component of fibrous connective tissue; also found in scar tissue.
Cytochrome: A class of enzymes that facilitates certain *oxidation* reactions.
Cytokine: A soluble molecule that regulates cellular interaction and cellular functions. Cytokines are produced and secreted by a variety of cells, including immune cells.
Eicosanoids: Physiologically active substances that affect liver function; some eicosanoids can protect the liver from damage, whereas others promote *hypoxia*, inflammation, and necrosis.
Endothelium: The layer of cells lining the inner wall of a blood vessel.
Endotoxin: A molecule in the cell wall of many bacteria in the intestine. Endotoxins are released into the body when the bacteria die and can cause fever, chills, shock, and other symptoms of infection.
Fibril: A very small threadlike structure (i.e., fiber) or a component of a fiber.
Fibrogenic: Conducive to the development of scar tissue.
Fibrogenesis: Development of scar tissue.
Fibronectin: A large adhesive extracellular matrix *glycoprotein.* Among its roles in the body, fibronectin is important in connective tissue, where it cross-links with *collagen*. A specialized subtype is capable of stimulating *stellate cells.*
Glutathione: A major *antioxidant.*
Glycoprotein: Compounds consisting of a protein and a carbohydrate group.
Growth factor: A *cytokine* that stimulates cell proliferation.
Hepatocyte: The principal cell type found in the liver; its many functions include bile production, protein synthesis, detoxification, and nutrient storage.
Hypoxia: Lower-than-normal oxygen concentrations.
Kupffer cells: Specialized immune cells (i.e., *macrophages*) in the liver that remove bacteria and other foreign organic substances from the blood.
Macrophage: An immune cell responsible for consuming foreign substances such as bacteria.
Matrix: The fundamental scaffolding of a tissue or organ in which specialized structures and cells are embedded.
Mitochondria: *Organelles* that generate energy (e.g., *ATP*) for the cell’s metabolic processes.
Organelle: The functional component of a cell; each organelle has its own membrane and specialized function.
Oxidation: A chemical reaction that removes a hydrogen atom from a substance or adds oxygen to it (or both).
Oxidative stress: An imbalance between oxidants and *antioxidants* leading to excessive oxidation and cell damage.
Portal triad: The three main channels interconnecting the liver’s functional units (i.e., lobules), consisting of tributaries from the hepatic artery and portal vein and a tributary leading to the common bile duct.
Proteoglycan: A specific group of large chemical substances found in connective tissue *matrix*, viscous lubricating body fluids (e.g., mucous secretions), and scar tissue. Proteoglycans contain a protein core with sugar (i.e., carbohydrate) side chains.
Reactive oxygen species: Highly reactive, oxygen-containing molecules that cannot exist in a free state for a prolonged period; also called oxygen radicals.
Sinusoids: Tiny blood vessels found in certain organs, including the liver, where they supply oxygen and nutrients to *hepatocytes*.
Space of Disse: The narrow space that separates *hepatocytes* from the *sinusoids* in the liver. (Also known as “subendothelial space.”)
Stellate cells: Star-shaped, droplet-containing cells residing in the *space of Disse* that serve as the primary storage site for vitamin A compounds in normal liver. Stellate cells (also known as lipocytes or as Ito, fatstoring, or perisinusoidal cells) are capable of producing scar protein when activated.
Superoxide: A destructive *reactive oxygen species* produced as a byproduct of some *oxidation* reactions.
